# Lack of Disease Specificity Limits the Usefulness of In Vitro Costimulation in HIV- and HCV-Infected Patients

**DOI:** 10.1155/2008/590941

**Published:** 2008-07-21

**Authors:** Stefanie Kuerten, Tobias R. Schlingmann, Tarvo Rajasalu, Doychin N. Angelov, Paul V. Lehmann, Magdalena Tary-Lehmann

**Affiliations:** ^1^Institut für Anatomie I, Medizinische Fakultät der Universität zu Köln, Joseph-Stelzmann-Str. 9, 50931 Köln, Germany; ^2^Department of Pathology, Case Western Reserve University, Wolstein Building, 10900 Euclid Avenue, Cleveland, OH 44106, USA

## Abstract

Measurements of antigen-specific T cell responses in chronic diseases are limited by low frequencies of antigen-specific cells in the peripheral blood. Therefore, attempts have been made to add costimulatory molecules such as anti-CD28 or IL-7/IL-15 to ELISPOT assays to increase sensitivity. While this approach has been successful under certain circumstances, results are often inconsistent. To date, there are no comprehensive studies directly comparing the in vitro effects of multiple costimulatory molecules in different disease settings. Therefore, in the present study we tested the effects of IL-7/IL-15, IFN-*α*, anti-ICOS, and anti-CD28 on antigen-specific T cell responses in patients infected with HCV or HIV versus healthy individuals. Our data show that none of the aforementioned molecules could significantly increase ELISPOT sensitivity, neither in HCV nor in HIV. Moreover, all of them caused false-positive responses to HCV and HIV antigens in healthy individuals. Our results question the broad use of in vitro costimulation.

## 1. INTRODUCTION

The measurement of immune responses to disease-specific antigens has become increasingly important in chronic viral infections. Particularly T cell responses have proven to be a valuable tool both for vaccine development and immune monitoring [1, 2]. Examining the phenotype and activation status of T cells (e.g., by FACS analysis) or their cytokine secretion profile (e.g., in ELISPOT assays) not only provides helpful information on the immunogenicity of individual viral antigens, but also reflects the patient's current state of immunocompetence, a vital piece of information for treatment and prognosis.

One major obstacle, however, is the intrinsically low-frequency and cytokine productivity of T cells in hosts suffering from chronic disease. Not only infections like HIV that directly impair T cell function, but also viral infections that target cells outside the immune system, such as HCV, have been shown to decrease T cell reactivity to viral antigensin vitro [3–10]. As shown by Yonkers et al., this effect is even more pronounced in cases of HIV/HCV coinfection [11]. This alteration of T cell function by chronic viral infections makes it almost impossible to accurately quantify the disease-specific T cell repertoire in vitro.

A great deal of hope has therefore been placed into the use of in vitro costimulation. Adding molecules such as agonistic anti-CD28 antibodies or cytokines like IL-7 and IL-15 to FACS and ELISPOT assays is intended to optimize the interaction between T cells and APC [11–13]. The rationale of a strong second signal compensating for a weak first signal and/or a high T cell activation threshold is indeed appealing. Pathogen-specific T cells would no longer escape detection due to suboptimal in vitro stimulation, but would be reliably activated above threshold and thus become detectable. Following this rationale, in vitro stimulation would allow for a precise quantification of each individual's antigen-specific T cell repertoire.

Several studies have indeed shown increased T cell responses after in vitro costimulation; Jennes et al. demonstrated that the presence of IL-7 and IL-15 during T cell stimulation in ELISPOT assays significantly increases the frequency of PPD-specific and CMV-specific T cells [12]. Calarota et al. later demonstrated that IL-15 alone suffices to enhance IFN-*γ* production to both simian-human immunodeficiency virus and HIV antigens in infected macaques [14]. Finally, Ott et al. demonstrated that agonistic anti-CD28 antibody increases the IFN-*γ* response to tetanus toxoid, mumps antigen, CMV, and EBV peptides in healthy individuals as well as responses to NS3 protein in HCV patients and proinsulin along with islet cell antigen in type 1 diabetes patients [13].

Other studies, however, indicate a rather limited effect of in vitro costimulation. As Yonkers et al. demonstrated, memory-effector CD8 cells show reduced responsiveness to CD28 costimulation in HCV/HIV coinfection [11]. Subudhi et al. carefully delineated the opposing effects of B7 family members on immune responses, and showed that immune modulators frequently lead to coinhibition rather than the desired stimulation [15].

Much uncertainty therefore remains mainly because no human study to date has systematically compared multiple types of in vitro costimulation in the setting of different viral infections. Moreover, most studies compare stimulated versus unstimulated responses within infected patients. Little attention, however, has so far been paid to the effect of in vitro costimulation in healthy, uninfected individuals. How specific are the responses obtained after costimulation? Is the desired increase in assay sensitivity compromised by decreased specificity?

Our present study sheds some light upon these essential questions. We systematically compared the effects of four different methods of in vitro costimulation: IL-7/IL-15, IFN-*α*, anti-ICOS, and anti-CD28. Using IFN-*γ* ELISPOT assays with and without costimulation, we measured the T cell response to disease-specific antigens in 10 HCV patients, 10 HIV patients, and 6 healthy individuals. Our results lead to three major conclusions. Firstly, in HCV patients, significant increases in T cell reactivity could only be seen with one particular antigen (NS3 protein) when using either IL-7/IL-15 or anti-CD28. Secondly, in HIV patients, none of the four types of in vitro costimulation led to a statistically significant response increase, regardless of the antigen used. Lastly and most importantly, all four methods of costimulation led to false-positive responses to HCV and HIV antigens in healthy individuals.

## 2. MATERIALS AND METHODS

### 2.1. Subjects and sample collection

Peripheral blood was obtained from 10 HIV positive and 10 HCV-infected subjects—the former from the Special Immunology Unit at University Hospitals of Cleveland, and the latter from the Cleveland Veterans Affairs Medical Center and University Hospitals of Cleveland. HIV infection was defined as a positive result for ELISA or other licensed HIV antibody tests, as well as previously detectable plasma HIV RNA. HCV infection was defined as detectable serum HCV antibodies and RNA. The six healthy controls tested were members of our and adjoining laboratories, and were negative for both HIV and HCV antibodies. For all participants, PBMCs were isolated from 40–100 mL of heparinized blood by standard Ficoll density-gradient centrifugation (IsoPrep, Robbins Scientific Corp., Sunnyvale, Calif, USA) and immediately used for ELISPOT analysis. All studies were performed under the approval of the Institutional Review Board for Human Investigation at the University Hospitals of Cleveland and the Cleveland Veterans Affairs Medical Center.

### 2.2. ELISPOT assays and image analysis

ImmunoSpot plates (Cellular Technology Ltd., Cleveland, Ohio, USA) were coated with IFN-*γ* capture antibody mAb M700A (Endogen, Woburn, Mass, USA) in PBS (3 *μ*g/mL) and placed at 4°C overnight. The plates were then blocked with PBS containing 1% BSA (Sigma-Aldrich, St. Louis, Mo, USA) for 1 hour and washed three times with PBS. Freshly isolated PBMCs were plated in RPMI-1640 (BioWhittaker, Walkersville, Md, USA) supplemented with 100 U/mL penicillin, 100 *μ*g/mL streptomycin, 2 mM L-glutamine, and 10% pooled heat-inactivated human AB serum. For all experiments, 3×10^5^ PBMCs were plated per well in the presence or absence of IL-7/IL-15 (final concentration of 5 ng/mL; Cell Sciences, Canton, Mass, USA), IFN-*α* (final concentration of 1000 U/mL; Biosource, Camarillo, Calif, USA), agonistic anti-ICOS antibody (final concentration of 1 *μ*g/mL; eBioscience, San Diego, Calif, USA), or agonistic anti-CD28 antibody (final concentration of 1 *μ*g/mL; BD Pharmingen, San Jose, Calif, USA). The following HIV antigen pools were used: Pool 1 (comprised of HIV-1 peptides A0301 *pol*, A201 *pol* 2, A24 *nef* 8, and A201 *nef* 1), Pool 2 (comprised of HIV-1 peptides A24 gp41, B27 gp120, A301 p17, and A201 *nef* 2), and Pool 3 (comprised of HIV-1 peptides B27 gp41, A201 *nef* 3, B51 *nef*, and A24 gp120). All peptide pools were used at 10 *μ*g/mL. All HIV peptides were obtained from the NIH AIDS Research and Reference Reagent Program, Division of AIDS, NIAID, NIH. Recombinant HCV core NS3 protein was supplied by Chiron (Emeryville, Calif, USA) and used at a concentration of 10 *μ*g/mL. A set of 73 MHC class I-restricted HCV peptides representing portions of the HCV genotype 1 protein sequence, based upon previously described CD8 determinants [16–19], were synthesized by the multipin technique (Chiron, Emeryville, Calif, USA.) and pooled together as a set (Pepset 4, used at 3.4 *μ*M). Phytohemagglutinin (PHA) was used as positive control in all assays, and was obtained from Sigma-Aldrich (10 *μ*g/mL). Negative control wells contained PBMC with a medium in the presence or absence of the costimulatory agents as mentioned above. After 24 hours of incubation at 37°C and 7% CO_2_, the plates were washed with PBS and PBS/TWEEN, and IFN-*γ* biotinylated detection antibody mAB M701 (Endogen, 2 *μ*g/mL) was added. The antibody was diluted in PBS containing 1% BSA and 0.025% TWEEN (Fisher Scientific International Inc., Hampton, NH, USA). The plates were incubated at 4°C overnight. Then they were washed three times with PBS/TWEEN, and subsequently streptavidin-HRP conjugate (DAKO, Carpinteria, Calif, USA) was added at 1/2000 dilution, incubated for 2 hours at room temperature, and removed by washing twice with PBS and PBS/TWEEN. The spots were visualized by adding HRP substrate 3-amino-9-ethylcarbozole (Pierce, Rockford, Ill, USA). The reaction was stopped by rinsing the plate with distilled water when distinct spots were visible macroscopically. Plates were dried overnight and images of the ELISPOT wells were captured with an ImmunoSpot Series 5 Analyzer (Cellular Technology Ltd.). Image analysis of the ELISPOT results was performed with the ImmunoSpot 5 Analysis Software (Cellular Technology Ltd.).

### 2.3. Statistical analysis

Mann-Whitney rank sum test was calculated using SigmaStat (Version 7; SPSS Inc., San Jose, Calif, USA) to test for significant differences between values obtained with and without the addition of IL-7/IL-15, IFN-*α*, anti-ICOS, or anti-CD28, respectively. A probability value of *P* ≤ .05 was considered to be statistically significant.

## 3. RESULTS

### 3.1. Characteristics of the study subjects

The clinical characteristics of the subjects evaluated for HCV- and HIV-specific T cell functions as well as of healthy individuals are listed in Table 1. Age and gender are listed for all subjects. For HCV-infected patients, HCV genotype, plasma HCV RNA levels, and platelet counts are given. Liver function is represented by serum albumin levels, ALT levels, and total bilirubin levels. For HIV-infected patients, CD4 cell counts and plasma HIV RNA levels are listed. The ratio of treated to untreated patients is given for both patient groups.

### 3.2. Increased responses to NS3 protein in HCV-infected patients only after in vitro costimulation with IL-7/IL-15 or anti-CD28

**Figure 1:**
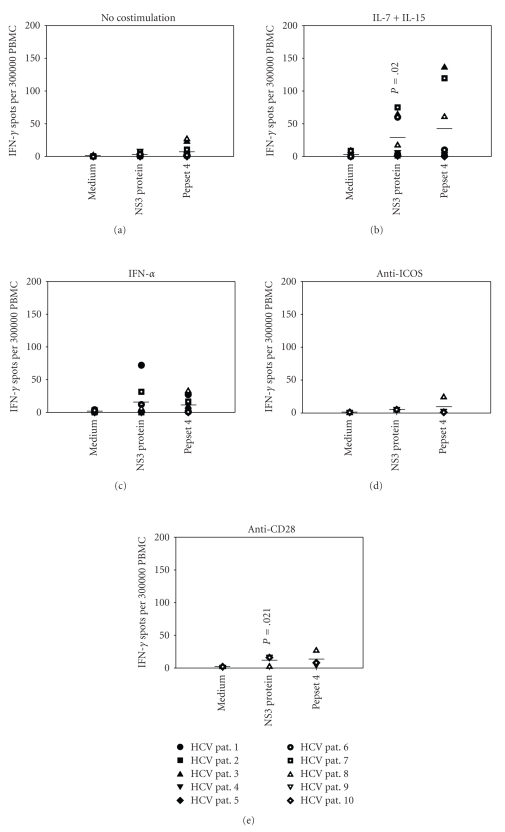
Increased responses to NS3 protein in HCV-infected patients only after in vitro costimulation with IL-7/IL-15 or anti-CD28. PBMCs of 10 HCV-infected patients were tested in IFN-*γ* ELISPOT assays for their recall responses to NS3 protein and Pepset 4 peptide pool in the absence (a) or presence of IL-7/IL-15 (b), IFN-*α* (c), anti-ICOS (d), or CD28 (e). For each individual, the mean spot count of duplicate wells is shown. Horizontal bars indicate the means of all individual responses to the respective antigens. *P* values are given for means that are significantly different from baseline values in (a). Symbols in (a) also apply to (b)–(e).

In order to compare the effects of different types of in vitro costimulation in 10 HCV-infected patients, we tested recall responses to NS3 protein and Pepset 4 peptide pool in IFN-*γ* ELISPOT assays. Results are shown in Figure 1.

Figure 1(a) illustrates the low baseline responses to NS3 protein and Pepset 4 pool (2.5 ± 2.5 and 6.8 ± 9.3 spots above medium, resp.) as frequently described in the literature in the case of chronic infection [3–10]. The presence of IL-7/IL-15 during in vitro T cell stimulation led to a significant ten-fold upregulation of IFN-*γ* recall responses to NS3 protein (Figure 1(b); *P* = .02). Three of the patients also showed increased responses to Pepset 4. However, due to the wide range of spot numbers, the mean increase was not statistically significant. Neither IFN-*α* (Figure 1(c)) nor anti-ICOS (Figure 1(d)) had a general effect on HCV-specific responses. Only HCV patients 1 and 7 showed an increased NS3 response upon IFN-*α* stimulation (*P* = .002 and *P* = .026, resp.). Costimulation with anti-CD28 (Figure 1(e)) showed similar results compared to IL-7/IL-15 with a significant four-fold increase of spot numbers (*P* = .021) after recall with NS3 protein. This is consistent with observations made by Yonkers et al. and Ott et al. showing that HCV-specific T cells remain responsive to CD28 stimulation during chronic infection [11, 13]. This consistency of results despite significantly different patient age distributions in the aforementioned studies also shows that patient age does not seem to influence the effects of in vitro costimulation.

### 3.3. In vitro costimulation has no effect on antigen-specific T cell responses in HIV-infected patients

**Figure 2:**
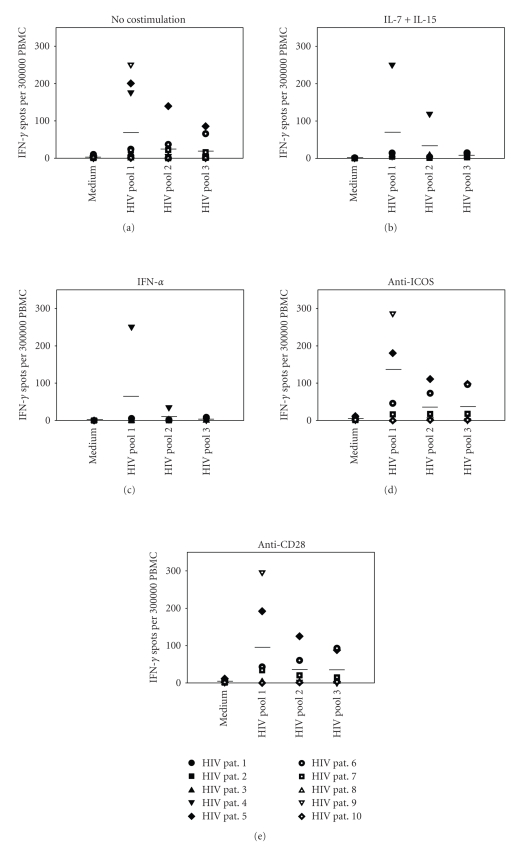
In vitro costimulation has no effect on antigen-specific T cell responses in HIV-infected patients. PBMCs of 10 HIV-infected patients were tested in IFN-*γ* ELISPOT assays for their recall responses to HIV peptide pools 1, 2, and 3 (as described in Section 2) in the absence (a) or presence of IL-7/IL-15 (b), IFN-*α* (c), anti-ICOS (d), or CD28 (e). For each individual, the mean spot count of duplicate wells is shown. Horizontal bars indicate the means of all individual responses to the respective antigens. Symbols in (a) also apply to (b)–(e).

In order to explore whether the aforementioned types of costimulation have a similar effect in the case of HIV infection, we tested recall responses to three different HIV peptide pools in 10 HIV-infected patients. Results are shown in Figure 2.

Unlike in HCV, some of the patients showed positive baseline responses to HIV peptides (Figure 2(a)). However, none of the costimulatory reagents could significantly enhance the overall response pattern, as shown in Figures 2(b)–2(e). None of the mean responses to any of the antigens was significantly elevated, *r*egardless of the (interindividually highly variable) baseline reactivity. Results were also identical in both antiretrovirally treated and untreated patients, showing that antiretroviral therapy did not seem to influence the effects of in vitro costimulation. This decreased responsiveness of HIV-infected lymphocytes to costimulation has previously been described by Borthwick et al. [20] and Trimble et al. [21], and is attributed to the downregulation of costimulatory molecules such as CD28 on T cells in the course of chronic HIV infection [22–24]. The precise mechanisms of this defect, however, are still not understood. Our data indicate that this effect may not be limited to CD28.

### 3.4. In vitro costimulation causes false-positive T cell responses to HCV and HIV antigens in healthy individuals

**Figure 3:**
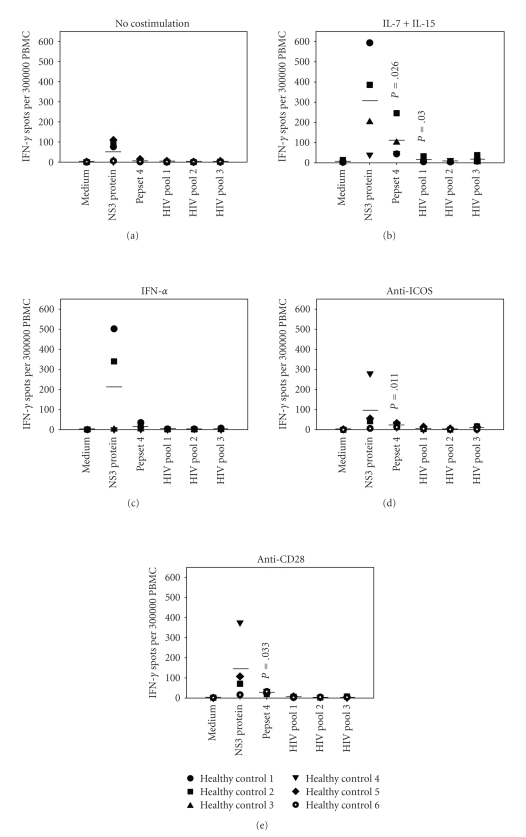
In vitro costimulation causes false-positive T cell responses to HCV and HIV antigens in healthy individuals. PBMCs of 6 healthy individuals were tested in IFN-*γ* ELISPOT assays for their recall responses to NS3 protein, Pepset 4 peptide pool, and HIV peptide pools 1, 2, and 3 in the absence (a) or presence of IL-7/IL-15 (b), IFN-*α* (c), anti-ICOS (d), or CD28 (e). For each individual, the mean spot count of duplicate wells is shown. Horizontal bars indicate the means of all individual responses to the respective antigens. *P* values are given for means that are significantly different from baseline values in (a). Symbols in (a) also apply to (b)–(e).

To date, there are no conclusive data available regarding the effect of in vitro costimulation on HCV- and HIV-specific recall responses in healthy individuals. This information is crucial, though, in order to be able to properly interpret positive test results obtained from assays using in vitro costimulation. Therefore, we specifically tested the same HCV and HIV antigens as above in 6 healthy individuals (Figure 3). If in vitro costimulation enhances existing responses without altering disease specificity, it should not induce positive responses in healthy individuals.

As shown in Figure 3, all four methods of in vitro costimulation (Figures 3(b)–3(e)) caused marked responses to HCV and/or HIV antigens in several healthy individuals. IL-7/IL-15 (Figure 3(b)) led to a strong positive response to Pepset 4 peptide pool (twenty-fold increase above baseline; *P* = .026) as well as to HIV pool 1 (ten-fold increase above baseline; *P* = .030). The mean frequency of NS3 reactive T cells was increased six-fold. Although this shift in mean frequency is not statistically significant due to the broad range of anti-NS3 responses, the changes for individual subjects are highly significant (*P* = .002 for Healthy Control 1; *P* = .005 for Healthy Control 2).

Similarly, IFN-*α* also induced false-positive responses to NS3 (Figure 3(c)), as seen in two individuals (*P* = .028 for Healthy Control 1; *P* = .021 for Healthy Control 2). Interestingly, these individuals had also displayed susceptibility to IL-7/IL-15 stimulation (Figure 3(b)). In vitro costimulation with anti-ICOS agonist (Figure 3(d)) upregulated anti-NS3 responses to a lesser degree. Healthy Control 4 responded with ~300 spots above baseline (*P* < .001). Remarkably, this individual had previously proven nonreactive to both IL-7/IL-15 and IFN-*α*. The mean frequency of Pepset 4 reactive T cells increased four-fold (*P* = .011) upon ICOS stimulation.

Finally, as shown in Figure 3(e), CD28 costimulation also caused false-positive test results. Healthy Control 4 responded to NS3 approximately three-fold above baseline (*P* = .006). Moreover, mean spot counts for Pepset 4 were also increased significantly (*P* = .033).

## 4. DISCUSSION

Efforts for in vitro T cell analysis attempt to establish frequencies of antigen-reactive cells and their effector functions, defined by the release of cytokines secreted upon antigen encounter. Tetramer staining for intracellular cytokines, cytolytic molecules, and antigen-specific T cell receptors (TCRs) is a technique frequently used for this purpose. It further allows the characterization of cell surface marker expression by antigen-specific cells, distinguishing, for example, between central and effector memory cells or between CD4 and CD8 cells [25].

In many cases, however, there is no true dominance of a particular peptide antigen. This has been described in HIV and recently also in response to vaccinia virus [26]. For this reason, immune diagnostics in infectious diseases is shifting towards the use of peptide pools as antigens. Since this approach is unfeasible in flow cytometry, functional test systems are commonly used for this purpose in which antigen directly activates the T cells and the resulting cytokine secretion is detected. Cytokines can be measured in the cell supernatant by ELISA or directly around the secreting cell using ELISPOT assays. ELISPOT assays typically are more sensitive compared to ELISA because they are able to detect the cytokine production of each individual cell before the cytokine is diluted into the supernatant, degraded by proteases, or neutralized by receptors of bystander cells. As far as cell frequencies are concerned, ELISPOT essentially provides the same information as intracellular cytokine staining (ICS). However, the detection limit of ELISPOT can be as low as 1/100 000 cells, ten times lower than ICS [27]. Moreover, ELISPOT measures the biologically relevant secretion of a cytokine as opposed to its intracellular storage detected by ICS. The intracellular presence of granzyme B or perforin, for example, merely indicates that the cell has encountered antigen within the past month [28]. In contrast, the actual release of granzyme B or perforin indicates immediate antigen recognition by a specific cell [1, 2].

All of these functional assays, however, have one major weakness in common. They provide accurate information on T cell frequencies and function only if all specific T cells present in the test cell population undergo full activation. For example, recognition of an antigen presented by a dendritic cell (DC) along with strong costimulation will result in full T cell activation. This lies in contrast with T cell activation by B cells which are known to be cells with rather weak costimulatory capabilities [29]. In the latter case, only a fraction of antigen-specific T cells will be fully activated. As a consequence, the assay will mainly detect those cells that happen to make contact with a DC instead of a B cell when sedimenting out of single-cell suspension onto the bottom of the test plate. Since DCs are less abundant in peripheral blood than B cells, the frequencies of antigen-specific T cells will be considerably underestimated. A T cell would acquire different effector functions depending on the type of APC presenting the antigen, and functional assays would be strongly influenced by the prevalent types of APC populations in the blood. Equally, conditions that lead to APC depletion or inactivation, such as HIV and cancer, would leave many antigen-specific T cells undetected, due to the lack of proper stimulation. This is one of the reasons why in chronic diseases such as HIV and HCV it has been notoriously difficult to detect antigen-specific T cells in significant numbers. Are T cells functionally impaired under such conditions or is their activation hampered by limited numbers of APC? This has not been sufficiently studied yet. For this reason, there have been increasing efforts in the field to include costimulatory signals in T cell activating cultures in order to overcome the deficiencies of the APC compartment or of the T cells themselves.

A prototypic costimulatory molecule is CD28 [30], the only B7 receptor constitutively expressed on naive T cells. In addition to ligation of TCR and MHC antigen complex, binding of CD28 to either B7.1 or B7.2 on the APC leads to upregulation of autocrine IL-2 secretion by the T cell. CD28 is commonly used for signal enhancement in flow cytometry. In a previous study, we found that it can also have “signal-enhancing” activity on recall responses to tetanus, mumps, CMV, and EBV antigens in healthy individuals.

In the experiments reported here, we extended our previous studies and tested the effects of different types of in vitro costimulation on HCV- and HIV-specific recall responses not only in HCV- and HIV-infected patients, but also in healthy individuals. Strikingly, we found that even in healthy donors anti-CD28 induced significant responses to Pepset 4 antigens in addition to a strong response to NS3 in one donor. While in HCV patients the results were weakly increased for NS3, in HIV patients CD28 costimulation did not result in signal enhancement to any of the antigens tested. Therefore, we conclude that the use of anti-CD28 agonist provides no consistent benefit in either HIV or HCV infection. In vitro costimulation with a combination of anti-CD28 and anti-CD49d antibodies led to similar results (data not shown).

We also tested the use of IFN-*α* as a costimulatory reagent. IFN-*α* constitutes a “danger signal” released by cells of the innate immune system when infected with virus or upon TLR stimulation [31]. In our study, IFN-*α* caused false-positive responses to NS3 in two of the healthy donors, while having little to no effect on responses to any of the other antigens. In HIV patients, the antigen-specific recall responses were even reduced in the presence of IFN-*α* while in HCV patients they were increased to NS3 protein in two of the patients. These data warrant further studies and clearly show that the use of IFN-*α* for in vitro costimulation can result in false-positive responses in healthy individuals while showing no useful effect in either HCV or HIV infection.

We also tested agonistic anti-ICOS antibody as a costimulatory reagent. Belonging to the CD28 family [30], anti-ICOS showed similar effects to anti-CD28. It did not have significant effects on recall responses in HIV and HCV patients, but caused false-positive responses to NS3 in healthy controls.

The combination of IL-7 and IL-15 has been used in in vitro assays to increase assay sensitivity [12]. Particularly for CD8 memory cells, both cytokines represent growth and differentiation factors. Strikingly, the combination of IL-7 and IL-15 induced strong responses in healthy controls to NS3 protein, Pepset 4, and HIV pool 1. Increased “antigen-specific” recall responses to both NS3 and Pepset 4 were also seen in HCV patients. However, in the light of the nonspecific stimulatory effect of IL-7/IL-15 in healthy controls, these responses cannot be interpreted as specific. Finally, similar to IFN-*α*, the combination of IL-7/IL-15 led to a marked reduction of cytokine responses to all three HIV peptide pools in HIV patients. It is unclear why HIV-infected individuals respond so differently compared to HCV patients and healthy controls. Possible reasons include the reduced T cell function and susceptibility to costimulation as a consequence of HIV infection as previously described for CD28 [22–24].

## 5. CONCLUSION

Overall, our data show that in vitro costimulation with IL-7/IL-15, IFN-*α*, anti-ICOS, and anti-CD28 is no “magic bullet” providing simple solutions for improved T cell diagnostics. There are two criteria that a method of in vitro costimulation has to fulfill in order to qualify as clinically and/or scientifically useful, reliably enhancing antigen responses in a disease-specific manner without causing false-positive responses in healthy individuals. Our results show that essentially none of the stimulatory molecules tested met these criteria. There are two possible interpretations of these results. On the one hand, insufficient costimulation may not be the limiting factor in HCV or HIV patients, which explains why additional costimulation does not help to reveal more specific activity. The other possibility is that the functional defects present in diseases like HIV and HCV are too severe—either on the T cell or the antigen presentation side—to be overcome by in vitro costimulation.

## Figures and Tables

**Table 1:** Clinical characteristics of study subjects. Data are given as means ± SD, unless otherwise specified. HCV: hepatitis C virus; HIV: human immunodeficiency virus; ALT: alanine aminotransferase; N/A: not applicable.

	HCV	HIV	Healthy
Gender	*n* = 10 males	*n* = 4 males	*n* = 4 males
		*n* = 6 females	*n* = 2 females
Age	52.75 ± 2.3	34.8 ± 5.9	29.6 ± 3.4
CD4 cell count, 10^6^ cells/L	N/A	451.3 ± 134.9	N/A
Plasma HIV RNA level, IU/mL	0.0 ± 0.0	8,685.1 ± 15,122.8	0.0 ± 0.0
HCV genotype	Type 1: *n* = 9	N/A	N/A
	Type 2: *n* = 1		
Plasma HCV RNA level, IU/mL	1,699,275 ± 1,505,121	0.0 ± 0.0	0.0 ± 0.0
Albumin, g/dL	3.9 ± 0.5	N/A	N/A
ALT, IU/L	80.3 ± 67.2	N/A	N/A
Total bilirubin level, mg/dL	0.5 ± 0.3	N/A	N/A
Platelet count, 10^3^ cells/mm^3^	211.6 ± 55.8	N/A	N/A
On treatment	4/10	8/10	0/10

## ABBREVIATIONS

AIDS: Acquired immunodeficiency syndrome
APC: Antigen presenting cell
BSA: Bovine serum albumin
CD: Cluster of differentiation
CMV: Cytomegalovirus
DC: Dendritic cell
EBV: Epstein-Barr virus
ELISA: Enzyme-linked immunosorbent assay
ELISPOT: Enzyme-linked immunosorbent spot
FACS: Fluorescent-activated cell sorting
HCV: Hepatitis C virus
HIV: Human immunodeficiency virus
HRP: Horseradish peroxidase
ICOS: Inducible costimulator
ICS: Intracellular cytokine staining
IFN: Interferon
IL: Interleukin
MHC: Major histocompatibility complex
NS3: Nonstructural HCV protein
PBMC: Peripheral blood mononuclear cell
PBS: Phosphate-buffered saline
PHA: Phytohemagglutinin
PPD: Purified protein derivative
RNA: Ribonucleic acid
RPMI: Roswell Park Memorial Institute
TCR: T cell receptor.
